# Diversity of Survival Patterns among *Escherichia coli* O157:H7 Genotypes Subjected to Food-Related Stress Conditions

**DOI:** 10.3389/fmicb.2016.00322

**Published:** 2016-03-15

**Authors:** Mohamed Elhadidy, Avelino Álvarez-Ordóñez

**Affiliations:** ^1^Department of Bacteriology, Mycology and Immunology, Faculty of Veterinary Medicine, Mansoura UniversityMansoura, Egypt; ^2^Teagasc Food Research CentreFermoy, Ireland

**Keywords:** *E. coli* O157:H7, genotypes, food, stress, survival

## Abstract

The purpose of this study was to evaluate the resistance patterns to food-related stresses of Shiga toxin producing *Escherichia coli* O157:H7 strains belonging to specific genotypes. A total of 33 *E*. *coli* O157:H7 strains were exposed to seven different stress conditions acting as potential selective pressures affecting the transmission of *E*. *coli* O157:H7 to humans through the food chain. These stress conditions included cold, oxidative, osmotic, acid, heat, freeze-thaw, and starvation stresses. The genotypes used for comparison included lineage-specific polymorphism, Shiga-toxin-encoding bacteriophage insertion sites, clade type, *tir* (*A255T*) polymorphism, Shiga toxin 2 subtype, and antiterminator *Q* gene allele. Bacterial resistance to different stressors was calculated by determining *D*-values (times required for inactivation of 90% of the bacterial population), which were then subjected to univariate and multivariate analyses. In addition, a relative stress resistance value, integrating resistance values to all tested stressors, was calculated for each bacterial strain and allowed for a ranking-type classification of *E. coli* O157:H7 strains according to their environmental robustness. Lineage I/II strains were found to be significantly more resistant to acid, cold, and starvation stress than lineage II strains. Similarly, *tir* (*255T*) and clade 8 encoding strains were significantly more resistant to acid, heat, cold, and starvation stress than *tir* (*255A*) and non-clade 8 strains. Principal component analysis, which allows grouping of strains with similar stress survival characteristics, separated strains of lineage I and I/II from strains of lineage II, which in general showed reduced survival abilities. Results obtained suggest that lineage I/II, *tir* (*255T*), and clade 8 strains, which have been previously reported to be more frequently associated with human disease cases, have greater multiple stress resistance than strains of other genotypes. The results from this study provide a better insight into how selective pressures encountered through the food chain may play a role in the epidemiology of STEC O157:H7 through controlling the transmission of highly adapted strains to humans.

## Introduction

Shiga toxin producing *Escherichia coli* (STEC) O157:H7 is a food-borne zoonotic pathogen that represents a major public health concern worldwide (Lee et al., 2011). Cattle are the primary reservoir of *E. coli* O157:H7 and the food chain is the predominant transmission route for outbreaks caused by this pathogen (Rangel et al., 2005; Grant et al., 2008). Outbreaks are commonly attributed to the consumption of contaminated meat, milk, and dairy products, particularly those derived from cattle (Griffin, 1995). Symptoms of infection include bloody diarrhea, vomiting, haemorrhagic colitis, and life-threatening sequelae, such as haemolytic uremic syndrome (HUS) (Rangel et al., 2005). Because of the potential complications of the infection by this pathogen and its low infective dose it is important to reduce the contamination throughout the food chain to low levels (Teunis et al., 2004).

During food processing (particularly in minimally processed foods or those processed using a hurdles technology approach), STEC O157:H7 encounter different stress conditions that might affect their fate along the food chain and therefore their transmission to humans. Moreover, STEC O157:H7 may develop adaptive responses to stress that may enable survival under more harsh conditions, enhancing resistance to subsequent processing conditions, and even impacting the disease-causing potential of bacterial strains and therefore the final outcome of the food-borne disease (Samelis and Sofos, 2003; Alvarez-Ordóñez et al., 2015). These stress conditions include (i) cold stress that occurs during food marketing and storage; (ii) oxidative stress that is induced in food systems by agents added to aid processing due to their powerful bactericidal effect; (iii) osmotic stress, mainly due to the use of salt as a common food preservative to control the growth of food spoilage and pathogenic bacteria; (iv) acid stress imposed by organic acids used to reduce the microbial load in foods or by the gastric acidity that represents the first line of the host innate defense following ingestion of contaminated food; (v) heat induced stress in food pasteurization and sterilization regimes that causes damage to bacterial proteins; (vi) Freeze-thaw cycles that disturb bacterial cells and cell aggregates through strong fluctuations in temperature; and (vii) starvation stress that occurs in the environment following nutrient deprivation.

Several population genetic studies have reported that, among *E. coli* O157:H7 strains, some bacterial genotypes exhibit significant variation in their relative frequency of isolation between the human and bovine host; in which a significantly less-diverse group of *E. coli* O157:H7 genotypes has been recovered from human clinical specimens. This was originally attributed to the possibility that only a subset of genotypes was involved in human infection and this subset would represent a minor subpopulation of the strains of bovine origin (Franz et al., 2012). This divergence is suggested to be crucial and advices a close tracking of clinical-biased strains that are more closely associated with human disease, likely have a high risk for virulence and transmission potential to humans (Franz et al., 2012; Mellor et al., 2013; Elhadidy et al., 2015a) and/or are more correlated with severe clinical symptoms (Manning et al., 2008; Elhadidy et al., 2015b). Whether the intermediate habitat (in particular the farm to fork chain) of *E. coli* O157:H7 plays a significant role in the shaping of clinical populations remains obscure. The lineage-specific polymorphism assay (LSPA-6) uses six genetic markers identified by octamer-based genome scanning to differentiate *E. coli* O157:H7 into three lineages (LI, LI/II, and LII) that exhibit phenotypic differences based on pathogenic potential and host specificity. Lineages I and II are recovered mainly from humans and bovines, respectively, while the intermediate lineage I/II has been less characterized regarding its host distribution (Ziebell et al., 2008; Zhang et al., 2010; Lee et al., 2011). Shiga toxin bacteriophage insertion (SBI) site analysis relies on amplification of the *stx* toxin genes (*stx*_1_ and *stx*_2_) and the insertion site junctions of their encoding bacteriophages, and discriminates *E. coli* O157:H7 strains based on their distribution, gene expression and virulence potential (Shaikh and Tarr, 2003; Besser et al., 2007). Clade typing is a typing method that uses 32 single nucleotide polymorphisms (SNPs) that can distinguish *E. coli* O157:H7 strains into nine distinct evolutionary clades, with clade 8 strains exhibiting more virulence and a closer association with clinical illness than strains of other clades (Manning et al., 2008). Moreover, *tir* (*A255T*) polymorphisms, Shiga toxin 2 subtype, and *stx_2_-*-specific Q antiterminator gene allele (located upstream of the prophage *stx_2_* region and responsible for expression levels of the *stx_2_* gene) have been also suggested as clinically relevant genetic markers among *E. coli* O157:H7 (Ahmad and Zurek, 2006; Bono et al., 2007; Persson et al., 2007).

The aim of this study was to evaluate whether the variations in transmission and/or virulence potential among *E. coli* O157:H7 strains might be attributed, at least in part, to variations in their resistance to adverse stress conditions encountered throughout the food chain through selecting well-adapted strains belonging to clinically relevant genotypes. In order to investigate this hypothesis, the behavior of 33 *E. coli* O157:H7 strains, isolated from a range of meat and dairy samples in Egypt and belonging to different specific genotypes, was monitored following their exposure to cold, oxidative, osmotic, acid, heat, freeze-thaw, and starvation stresses, and the relationships between stress resistance patterns and genotypes were further analyzed by univariate and multivariate methods.

## Materials and Methods

### Bacterial Strains and Genetic Characterization

A total of 33 *E. coli* O157:H7 strains isolated during a previous study (Elhadidy and Elkhatib, 2015) from various food sources, including different meat and dairy samples, were used in this study. The meat samples included retail minced beef, hamburgers, and fresh beef samples. The dairy samples included raw milk and raw milk cheese samples. All bacterial strains were characterized using different genotyping methods as described in the previous study by Elhadidy and Elkhatib (2015). Genetic characterization of the strains included: lineage-specific polymorphism assay (LSPA-6) (Ziebell et al., 2008; Zhang et al., 2010), Shiga-toxin-encoding bacteriophage insertion site assay (SBI) (Shaikh and Tarr, 2003), clade typing (Manning et al., 2008), *tir* (*A255T*) polymorphism analysis (Bono et al., 2007), *stx_2_* subtyping (*stx*_2a_ and *stx*_2c_) (Persson et al., 2007), and antiterminator *Q* gene allele (*Q*_933_ and *Q*_21_) analysis (Ahmad and Zurek, 2006).

### Bacterial Culture Conditions and Stress Treatments

Bacterial strains were stored at -80°C using Pro-Lab Microbank cryovials (Pro-Lab, Richmond Hill, ON, Canada) according to the manufacturer’s instructions. Strains were cultured from frozen stocks onto Tryptone Soy Agar (TSA; Oxoid Ltd, UK) and incubated aerobically at 37°C for 24 h for their recovery before use in stress challenge experiments. All strains were subjected to seven different stress conditions commonly encountered during food processing and storage. One isolated colony from each tested *E. coli* O157:H7 strain was aseptically inoculated into 10 ml of TSB and incubated for 16 h at 37°C. This suspension was aseptically inoculated to 40 ml of sterile TSB (1:5 dilution), followed by incubation at 37°C for 24 h, which results in a stationary phase culture with approximately 10^9^ cells/ml, as described by Alvarez-Ordóñez et al. (2013). Actual starting bacterial numbers were confirmed by plating serial dilutions on TSA before applying the stressor. For all stress conditions, each stationary-phase culture was centrifuged at 7,500 × *g* for 7 min. The supernatant liquid was removed and cellular pellets were resuspended in 5 ml of sterile TSB (except for starvation stress in which the pellets were suspended in 0.85% saline). These bacterial suspensions were subjected to different stress conditions including: chilling to 5°C (cold stress) for up to 7 days, heating in a water bath at 55°C (heat stress) for up to 6 h, exposure to TSB with 1 mM H_2_O_2_ prewarmed to 37°C (oxidative stress) for up to 6 h, exposure to TSB with 5% (wt/vol) NaCl prewarmed at 37°C (osmotic stress) for up to 7 days, and exposure to TSB at pH 2.5 (adjusted with hydrochloric acid) prewarmed to 37°C (acid stress) for up to 6 h. For freeze-thaw stress, bacterial suspensions were subjected to seven cycles of freezing at -20°C for 22.5 h followed by thawing at 37°C for 1.5 h (each cycle lasted for 1 and 7 days were required to complete seven cycles). Finally, for starvation stress, bacterial pellets were resuspended in saline solution (0.85% NaCl, pH 6.6) and incubated at 37°C for up to 7 days. During each stress treatment, 0.1 ml of bacterial suspension was removed at set time intervals, serially 10-fold diluted in 0.1% peptone water and plated in triplicate on TSA to estimate the mean number of CFU/ml. The set time intervals for heat, oxidative, and acid stress were 0, 2, 4, and 6 h post-treatment. For freeze-thaw, cold, osmotic, and starvation stresses, the set time intervals were days 0, 2, 4, and 7. Three independent trials were carried out for each strain and stress condition.

### Determination of Stress Resistance Parameters

*D*-values, defined as the time required (in h for heat, oxidative, and acid stress or in days for freeze-thaw, cold, osmotic, and starvation stresses) for inactivation of 90% of the bacterial population, were determined by plotting the log_10_ number of survivors against time. The line that best fitted survivor plots was determined by linear regression (GraphPad Prism version 4.00 for Windows. GraphPad Software. San Diego, CA, USA) and the negative reciprocal of the slope was used for *D*-value determinations.

### Assignement of Relative Stress Resistance Units

An arbitrary unit was assigned for each *E. coli* O157:H7 strain under each stress condition by calculating the ratio between the *D*-value estimated for the particular strain subjected to a given stress and the maximum *D*-value observed among the collection of *E. coli* O157:H7 strains for that particular stress condition. Arbitrary units calculated for each stress condition were added to calculate a relative stress resistance value for each bacterial strain (ranging from 0 to 7), which was considered indicative of the general environmental robustness of the strain.

### Univariate Analyses of STEC O157:H7 Stress Resistance

*D*-values of strains from different genotypes [resulting form analysis of lineage-specific polymorphisms, Shiga-toxin-encoding bacteriophage insertion sites, clade typing, *tir* (*A255T*) polymorphisms, Shiga toxin 2 subtype, and antiterminator *Q* gene allele] were compared using Student’s *t*-test and the Statistica for Windows v 7.0. program (Statsoft, Inc., Tulsa, OK, USA).

### Multivariate Analysis of *E. coli* O157:H7 Stress Resistance

To gain insight into patterns of stress resistance, *D*-values from all of the seven stress resistance assays were analyzed by principal component analysis (PCA) as described elsewhere (Lee et al., 2012). PCA is useful for identifying a trend in a multivariate data set or a correlation between variables. The transformation of PCA consolidates the information of a data set into a few new variables or principal components (PCs). All of the analyses (calculation of coefficients, joining of variables, canonical analysis, and graphical display) were carried out with the Statistica for Windows, v 7.0. program.

## Results

### Inactivation of *E. coli* O157:H7 Strains by Different Stressors

The resistance to seven stressors (cold, oxidative, osmotic, acid, heat, freeze-thaw, and starvation) of 33 STEC O157:H7 strains was monitored over time, what allowed the study of the inactivation kinetics. Survival curves obtained for all stress conditions fitted properly into a first order kinetic. The goodness of fit was determined both by visual inspection and *R*^2^ value which ranged from 0.83 to 0.99 (in most cases *R*^2^ values of over 0.95 were observed; data not shown). *D*-values calculated are shown in **Table 1** as mean values and standard deviation.

**Table 1:** *D*-values for each strain and stress condition expressed in hours (acid, heat, and oxidative stress) and in days (freeze/thaw, cold, osmotic, and starvation stress).

Strain	Acid (h)^∗^	Heat (h)	Oxidative (h)	Freeze/thaw (d)	Cold (d)	Osmotic (d)	Starvation (d)
ESC22	5.42 ± 0.32	10.83 ± 1.31	26.87 ± 2.68	2.18 ± 0.21	1.99 ± 0.18	2.08 ± 0.15	1.79 ± 0.10
ESC469	7.71 ± 0.80	9.04 ± 0.56	21.83 ± 2.16	2.13 ± 0.22	2.10 ± 0.18	2.14 ± 0.07	2.21 ± 0.06
ESC323	18.32 ± 2.02	14.66 ± 1.93	19.33 ± 1.58	2.25 ± 0.19	2.11 ± 0.20	1.95 ± 0.15	2.29 ± 0.13
ESC412	16.11 ± 2.35	11.45 ± 1.43	22.29 ± 1.77	2.16 ± 0.20	2.197 ± 0.21	2.05 ± 0.16	2.21 ± 0.10
ESC154	5.55 ± 0.44	13.47 ± 1.65	14.46 ± 2.12	2.05 ± 0.18	2.30 ± 0.19	2.11 ± 0.20	2.31 ± 0.14
ESC123	12.90 ± 0.98	8.38 ± 0.97	11.17 ± 0.77	2.08 ± 0.22	2.22 ± 0.21	2.26 ± 0.07	2.37 ± 0.13
ESC415	12.77 ± 1.31	15.1 ± 1.44	14.70 ± 1.56	2.12 ± 0.17	2.28 ± 0.24	2.12 ± 0.17	2.30 ± 0.10
ESC76	4.38 ± 0.41	8.93 ± 1.20	21.02 ± 2.99	2.05 ± 0.19	2.18 ± 0.21	2.19 ± 0.15	1.89 ± 0.14
ESC91	4.06 ± 0.33	8.75 ± 0.79	19.15 ± 2.52	1.97 ± 0.10	2.24 ± 0.20	2.07 ± 0.14	1.93 ± 0.15
ESC10	6.72 ± 0.79	17.94 ± 1.68	12.82 ± 0.80	2.06 ± 0.15	2.28 ± 0.24	2.33 ± 0.07	2.30 ± 0.10
ESC19	6.69 ± 0.88	16.75 ± 1.99	10.77 ± 0.92	1.96 ± 0.08	2.18 ± 0.19	1.93 ± 0.15	2.16 ± 0.12
ESC378	9.19 ± 0.96	28.92 ± 5.09	6.80 ± 0.64	2.12 ± 0.16	2.32 ± 0.17	2.20 ± 0.15	2.44 ± 0.16
ESC113	6.11 ± 0.49	8.13 ± 1.11	7.92 ± 0.97	2.27 ± 0.18	2.15 ± 0.24	2.08 ± 0.04	2.27 ± 0.10
ESC227	6.37 ± 0.65	6.63 ± 0.89	5.64 ± 0.33	2.13 ± 0.21	2.20 ± 0.15	2.08 ± 0.09	2.26 ± 0.14
ESC318	4.53 ± 0.54	14.33 ± 1.44	6.79 ± 0.61	2.34 ± 0.18	2.25 ± 0.19	2.26 ± 0.17	2.40 ± 0.10
ESC65	6.30 ± 0.49	8.85 ± 1.05	11.98 ± 0.81	2.29 ± 0.07	2.25 ± 0.13	1.87 ± 0.18	2.27 ± 0.09
ESC51	5.55 ± 0.40	6.67 ± 0.90	6.51 ± 0.69	1.10 ± 0.09	2.22 ± 0.12	2.00 ± 0.07	2.06 ± 0.10
ESC63	10.40 ± 1.12	11.19 ± 0.86	17.45 ± 1.58	2.00 ± 0.11	2.08 ± 0.24	2.13 ± 0.22	2.25 ± 0.13
ESC70	9.42 ± 0.82	15.74 ± 1.14	8.95 ± 0.81	2.54 ± 0.24	2.33 ± 0.18	2.15 ± 0.11	2.33 ± 0.10
ESC83	5.83 ± 0.75	9.35 ± 0.79	9.78 ± 1.02	2.06 ± 0.13	2.30 ± 0.17	2.02 ± 0.12	2.40 ± 0.13
ESC347	6.99 ± 0.80	15.56 ± 1.51	9.76 ± 1.20	2.24 ± 0.26	2.25 ± 0.20	2.11 ± 0.19	2.57 ± 0.24
ESC98	5.86 ± 0.31	9.39 ± 1.14	14.28 ± 1.33	2.08 ± 0.11	2.19 ± 0.08	2.00 ± 0.22	1.74 ± 0.11
ESC120	13.72 ± 1.80	14.59 ± 1.35	17.30 ± 2.17	1.93 ± 0.19	2.16 ± 0.12	2.09 ± 0.19	2.49 ± 0.11
ESC395	9.09 ± 0.86	7.01 ± 0.52	8.03 ± 0.75	2.19 ± 0.19	2.19 ± 0.27	2.16 ± 0.21	2.21 ± 0.09
ESC367	15.17 ± 1.94	25.3 ± 2.76	11.05 ± 0.93	2.24 ± 0.16	2.11 ± 0.27	2.08 ± 0.13	2.68 ± 0.14
ESC473	3.77 ± 0.34	7.08 ± 0.44	7.19 ± 0.91	2.38 ± 0.14	2.09 ± 0.27	2.15 ± 0.09	1.76 ± 0.10
ESC563	15.36 ± 1.39	8.81 ± 0.85	7.93 ± 0.96	2.09 ± 0.11	2.25 ± 0.12	2.29 ± 0.08	2.40 ± 0.06
ESC190	10.08 ± 0.86	5.41 ± 0.63	7.55 ± 0.65	2.10 ± 0.21	2.34 ± 0.18	2.09 ± 0.12	2.31 ± 0.06
ESC115	5.58 ± 0.54	8.73 ± 1.14	9.01 ± 0.64	1.96 ± 0.05	2.28 ± 0.27	2.06 ± 0.15	2.42 ± 0.17
ESC175	5.62 ± 0.42	7.23 ± 0.81	14.65 ± 1.45	1.60 ± 0.21	2.08 ± 0.09	1.94 ± 0.17	1.77 ± 0.14
ESC193	10.49 ± 1.32	11.97 ± 1.50	12.98 ± 1.16	2.01 ± 0.19	2.14 ± 0.17	1.96 ± 0.18	2.36 ± 0.08
ESC657	10.34 ± 1.11	16.26 ± 2.18	8.54 ± 1.05	2.21 ± 0.15	2.22 ± 0.26	2.02 ± 0.21	2.31 ± 0.28
ESC470	5.59 ± 0.25	6.12 ± 0.38	23.65 ± 2.80	2.85 ± 0.22	1.88 ± 0.12	2.27 ± 0.30	1.81 ± 0.19


*h, in hours; d, in days.

### Univariate Analysis of Differences in Resistance to Different Stressors among *E. coli* O157:H7 Genotypes

**FIGURE 1:**
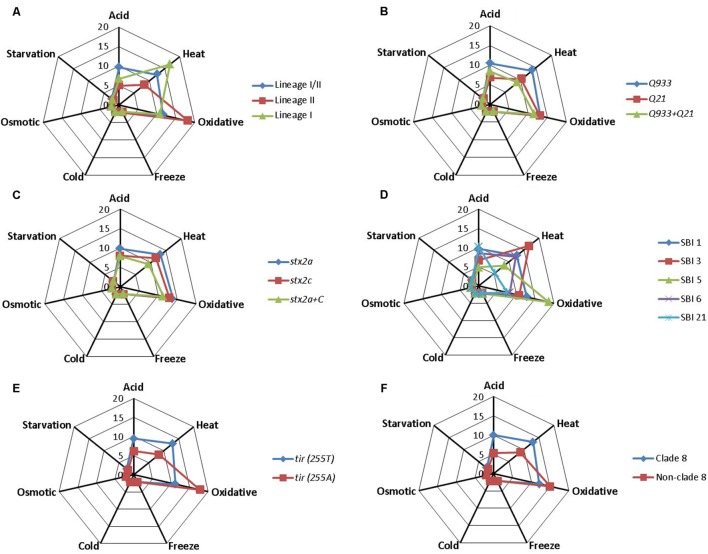
**Mean *D*-values in h (for acid, heat, and oxidative stress) or days (cold, osmotic, freeze-thaw, and starvation stress) for each *Escherichia coli* O157:H7 genotype.** **(A)** Lineage-specific polymorphisms; **(B)** antiterminator *Q* gene allele; **(C)** Shiga toxin 2 subtype; **(D)** Shiga-toxin-encoding bacteriophage insertion sites; **(E)** *tir* (*A255T*) polymorphisms; **(F)** clade typing.

**FIGURE 2:**
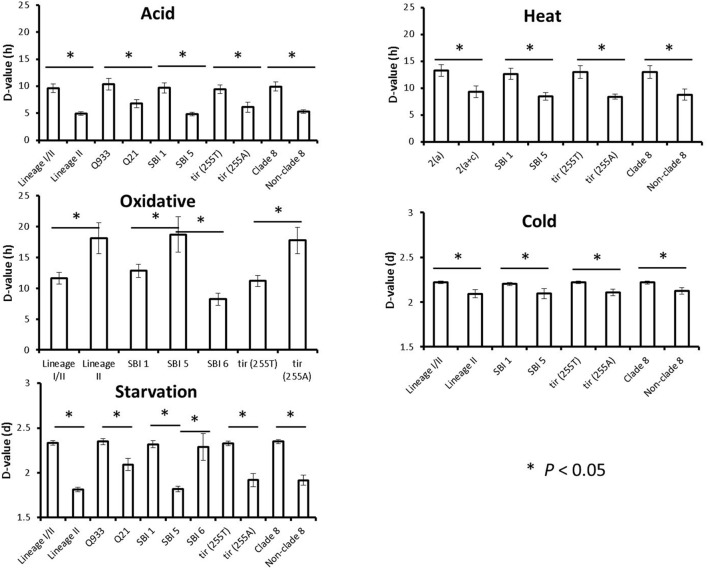
**Genotype-associated statistically significant differences in resistance to acid, heat, oxidative, cold, and starvation stresses.** Means and standard errors of the mean are shown.^∗^*P* < 0.05.

Associations between estimated *D*-values and singular STEC O157:H7 genotypes were evaluated individually for each stress condition by using the Student’s *t-*test. Mean *D*-values obtained for each particular genotype and stress condition are represented in **Figure 1**, while significant differences (*P* < 0.05) in stress resistance among different genotypes are shown in **Figure 2**. Statistically significant differences among genotypes shown in **Figure 2** refer in all cases to genotypes for which at least five representative strains were available in the strain collection. Lineage I/II strains, with mean *D*-values of 9.62 h, 2.22 and 2.33 days, respectively, were significantly more resistant to acid, cold, and starvation stressors than lineage II strains, with mean *D*-values of 4.96 h, 2.09 and 1.81 days. On the other hand they were significantly less resistant to oxidative stress, with a mean *D*-value of 11.62 h (vs. 18.12 h for lineage II strains). Only one lineage I strain was included in the study and showed *D*-values of 6.69 h, 10.77 h, 2.18 days and 2.16 days for acid, oxidative, cold and starvation treatments, respectively. *Q*_933_ strains were significantly more resistant to acid and starvation stress (*D*-values of 10.41 h and 2.35 days) than *Q*_21_ strains (*D*-values of 6.81 h and 2.09 days). Strains carrying *stx*_2a_ subtype were significantly more resistant to heat stress than strains carrying both *stx*_2a_ and *stx*_2c_ subtypes (mean *D*-value of 13.29 h vs. 9.32 h). SBI genotype 1 strains were significantly more resistant to acid, heat, cold, and starvation stress than SBI genotype 5 strains (SBI genotype 6 strains were also more resistant than SBI genotype 5 strains for starvation stress). On the contrary, both SBI genotypes 1 and 6 were significantly more sensitive to oxidative stress than SBI genotype 5 strains. Regarding *tir* (*A255T*) polymorphisms, *tir* (*255T*) encoding strains, with mean *D*-values of 9.44 h, 13.03 h, 2.22 days and 2.33 days, respectively, were significantly more resistant to acid, heat, cold, and starvation stressors than *tir* (*255A*) encoding strains, with mean *D*-values of 6.14 h, 8.42 h, 2.11 days and 1.92 days. On the other hand they were significantly less resistant to oxidative stress, with a mean *D*-value of 11.18 h [vs. 17.76 h for *tir* (*255A*) encoding strains]. The same trends described for *tir* (*A255T*) polymorphisms were observed for clade typing, with clade 8 strains being significantly more resistant to acid, heat, cold, and starvation stresses and more sensitive to oxidative stress than non-clade 8 strains. For osmotic and freeze-thaw stresses, no genotype-associated statistically significant differences in stress resistance patterns were found.

### Multivariate Analysis of *E. coli* O157:H7 Stress Resistance

**FIGURE 3:**
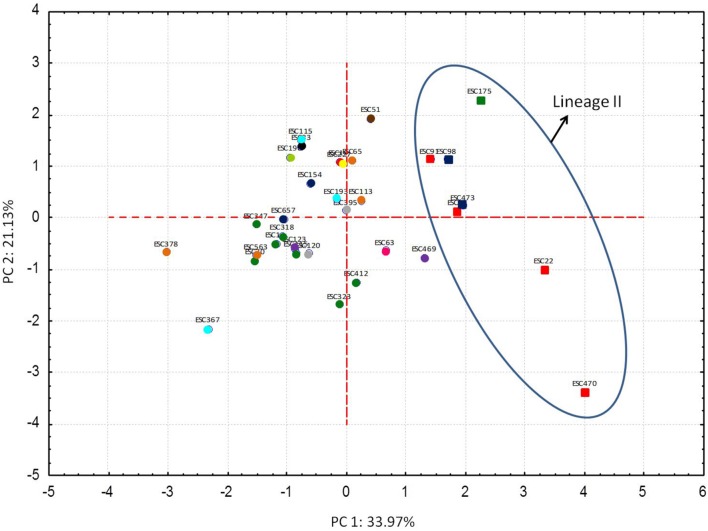
**Results of principal component analysis (PCA).** Distances between data points represent the similarities between strains. Each STEC O157:H7 genotype corresponds to a color, as shown below. 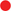 Lineage I, Q_933_, *stx_2a_*, SBI 3, *tir(255T)*, non-clade 8 strains; 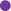 Lineage I/II, Q_933_, *stx_2a_*, SBI 1, *tir(255A)*, clade 8 strains; 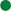 Lineage I/II, Q_933_, *stx_2a_*, SBI 1, *tir(255T)*, clade 8 strains; 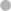 Lineage I/II, Q_933_, *stx_2a+c_*, SBI 1, *tir(255T)*, clade 8 strains; 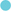 Lineage I/II, Q_933_, *stx_2c_*, SBI 1, *tir(255T)*, clade 8 strains; 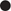 Lineage I/II, Q_21_, *stx_2a+c_*, SBI 1, *tir(255T)*, clade 8 strains; 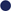 Lineage I/II, Q_21_, *stx_2c_*, SBI 1, *tir(255T)*, clade 8 strains; 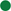 Lineage I/II, Q_21_, *stx_2c_*, SBI 6, *tir(255T)*, clade 8 strains; 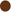 Lineage I/II, Q_21_, *stx_2c_*, SBI 6, *tir(255T)*, non-clade 8 strains; 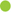 Lineage I/II, Q_21_, *stx_2c_*, SBI 21, *tir(255T)*, clade 8 strains; 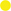 Lineage I/II, Q_933_+Q_21_, *stx_2a+c_*, SBI 1, *tir(255T)*, non-clade 8 strains; 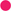 Lineage I/II, Q_933_+Q_21_, *stx_2a+c_*, SBI 1, *tir(255T)*, clade 8 strains; 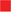 Lineage II, Q_21_, *stx_2c_*, SBI 5, *tir(255A)*, non-clade 8 strains; 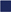 Lineage II, Q_21_, *stx_2a+c_*, SBI 5, *tir(255A)*, non-clade 8 strains; 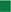 Lineage II, Q_21_, *stx_2a_*, SBI 1, *tir(255A)*, non-clade 8 strains.

In order to carry out a global analysis of STEC O157:H7 stress resistance, in first place, a relative stress resistance value that was considered indicative of the general robustness of the strain was calculated for each strain (**Table 2**). The fourteen more robust strains, according to their relative stress resistance value, were of lineage I/II and clade 8. On the other hand the five more stress sensitive strains were non-clade 8 strains. In second place, resistance parameters (*D*-values) were included in a multivariate PCA. The first and second PCs accounted for 33.97 and 21.13%, respectively, of the variance within the multivariate data set (**Figure 3**). Since most of the variation (55.1%) was contained in the first and second PC, we focused on these PCs. PCA allowed the identification of genotype-associated patterns of stress resistance. Thus, it clustered together all strains of lineage II [all of them *Q*_21_, *tir* (*255A*), non-clade 8 strains], that therefore had similar stress resistance patterns, and differentiated them from lineage I/II and lineage I strains. The factor loadings on the first PC (**Supplementary Table S1**) were negative values for acid, heat, cold, osmotic, and starvation stresses. Higher negative scores on the first PC were associated with greater resistance to these five stresses, and positive scores on the first PC were associated with susceptibility to them. Thus, resistance to these stresses was to some extent positively co-related and associated with sensitivity to oxidative and freeze-thaw stresses, which showed positive values for the factor loadings on the first PC. Pairwise comparisons of PC scores in the first PC and STEC O157:H7 genotypes evidenced that strains of lineages I and I/II, *Q*_933_, *stx*_2a_, SBI genotypes 1, 3, 6, and 21, *tir* (*255T*), and clade 8, which showed negative PC scores in the first PC, were in general more resistant to acid, heat, cold, and starvation stresses, and more sensitive to oxidative stress than their counterparts (**Supplementary Table S2**).

**Table 2:** Genotypic characteristics and relative stress resistance for the tested strains.

Strain	Relative stress resistance	Lineage	*Q*_933_/*Q*_21_	*stx_2_* subtype	SBI Genotype	*tir* (*A255T*)	Clade type
ESC367	5.69	I/II	*Q*_933_	*stx*_2c_	1	*tir* (*255T*)	8
ESC323	5.61	I/II	*Q*_933_	*stx*_2a_	1	*tir* (*255T*)	8
ESC412	5.51	I/II	*Q*_933_	*stx*_2a_	1	*tir* (*255T*)	8
ESC378	5.34	I/II	*Q*_21_	*stx*_2c_	6	*tir* (*255T*)	8
ESC120	5.29	I/II	*Q*_933_	*stx*_2a+C_	1	*tir* (*255T*)	8
ESC415	5.23	I/II	*Q*_933_	*stx*_2a_	1	*tir* (*255T*)	8
ESC70	5.07	I/II	*Q*_933_	*stx*_2a_	1	*tir* (*255T*)	8
ESC10	5.02	I/II	*Q*_933_	*stx*_2a_	1	*tir* (*255T*)	8
ESC563	5.02	I/II	*Q*_21_	*stx*_2c_	6	*tir* (*255T*)	8
ESC123	4.95	I/II	*Q*_933_	*stx*_2a_	1	*tir* (*255A*)	8
ESC63	4.94	I/II	*Q*_933_+*Q*_21_	*stx*_2a+C_	1	*tir* (*255T*)	8
ESC469	4.94	I/II	*Q*_933_	*stx*_2a_	1	*tir* (*255A)*	8
ESC347	4.90	I/II	*Q*_933_	*stx*_2a_	1	*tir* (*255T*)	8
ESC657	4.89	I/II	*Q*_21_	*stx*_2c_	1	*tir* (*255T*)	8
ESC470	4.85	II	*Q*_21_	*stx*_2c_	5	*tir* (*255A*)	Non 8
ESC22	4.85	II	*Q*_21_	*stx*_2c_	5	*tir* (*255A*)	Non 8
ESC193	4.81	I/II	*Q*_933_	*stx*_2c_	1	*tir* (*255T*)	8
ESC154	4.78	I/II	*Q*_21_	*stx*_2c_	1	*tir* (*255T*)	8
ESC318	4.64	I/II	*Q*_933_	*stx*_2a_	1	*tir* (*255T*)	8
ESC76	4.63	II	*Q*_21_	*stx*_2c_	5	*tir* (*255A*)	Non 8
ESC19	4.60	I	*Q*_933_	*stx*_2a_	3	*tir* (*255T*)	Non 8
ESC65	4.51	I/II	*Q*_21_	*stx*_2c_	6	*tir* (*255T*)	8
ESC190	4.51	I/II	*Q*_21_	*stx*_2c_	21	*tir* (*255T*)	8
ESC91	4.50	II	*Q*_21_	*stx*_2c_	5	*tir* (*255A*)	Non 8
ESC395	4.49	I/II	*Q*_933_	*stx*_2a+C_	1	*tir* (*255T*)	8
ESC83	4.47	I/II	*Q*_21_	*stx*_2a+C_	1	*tir* (*255T*)	8
ESC115	4.39	I/II	*Q*_933_	*stx*_2c_	1	*tir* (*255T*)	8
ESC113	4.36	I/II	*Q*_21_	*stx*_2c_	6	*tir* (*255T*)	8
ESC98	4.35	II	*Q*_21_	*stx*_2a+C_	5	*tir* (*255A*)	Non 8
ESC227	4.21	I/II	*Q*_933_+*Q*_21_	*stx*_2a+C_	1	*tir* (*255T*)	Non 8
ESC51	4.05	I/II	*Q*_21_	*stx*_2c_	6	*tir* (*255T*)	Non 8
ESC175	4.04	II	*Q*_21_	*stx*_2a_	1	*tir* (*255A*)	Non 8
ESC473	4.02	II	*Q_21_*	*stx*_2c_	5	*tir* (*255A*)	Non 8


*Strains are ordered by their relative stress resistance.*

## Discussion

The high virulence of STEC has stimulated interest in their determinants of survival in food and the environment. STEC encounter various environmental stresses in their ecological niches, in the environment of food-processing industries, on foods and in the host after their ingestion. These include fluctuations of pH, osmolarity, temperature, and oxygen availability, among others. In addition, industrial food preservation regimes commonly rely upon imposing extreme physical and chemical stresses with the aim to inactivate or limit the growth of pathogenic bacteria. Thus, a variety of preservation technologies (including thermal processing) impose a challenge to bacterial cells and can determine their fate along the food chain (Alvarez-Ordóñez et al., 2015).

In this study, the relationship between specific genotypes of STEC O157:H7 and stress resistance was assessed by using univariate and multivariate analyses. Different patterns of stress resistance in STEC O157:H7 tested isolates were observed after exposure to multiple food-related stressors: cold, oxidative, osmotic, acid, heat, freeze-thaw, and starvation stresses. When stress resistance parameters (*D*-values) were compared among STEC O157:H7 genotypes, significant differences were observed for acid, heat, oxidative, cold, and starvation stresses. Interestingly, lineage I/II, *tir* (*255T*), and clade 8 encoding strains, which showed a significantly higher resistance to acid, cold, and starvation stresses, have been shown in different studies to be more virulent and more frequently associated with human infection than their counterparts (Bono et al., 2007; Manning et al., 2008; Elhadidy et al., 2015b). On the contrary, some of these genotypes more commonly associated with human disease were significantly less resistant to oxidative stress. Although there is a controversy on whether bacterial responses to environmental stresses can modulate virulence (Archer, 1996; Gahan and Hill, 1999), it is documented that some genetic systems involved in specific stress responses are the same as those associated with virulence during infection. Indeed, some stress response regulators (e.g., *rpoS* in Gram-negatives, σ^B^ in Gram-positives) are known to be involved in the regulation of pathogenicity traits of certain food-borne pathogens, which suggests that stress responses may be an important factor in potentiating the expression of particular virulence factors *in vivo* (Hengge-Aronis, 2000). Thus, the ability to deal successfully with environmental stresses would indirectly help bacterial virulence (Alvarez-Ordóñez et al., 2015). Genes needed to withstand stress conditions in the environment would help bacteria to access the gastrointestinal interface and eventually provoke virulence in the host, with both stress-related and virulence-related genes being expressed in response to surrounding signals.

Several research groups have dedicated efforts in the last decade to evaluate the heterogeneity in STEC stress resistance, using both laboratory domesticated strains and field isolates (Saridakis et al., 2004; Bhagwat et al., 2006; Malone et al., 2007; Vanaja et al., 2009; Lee et al., 2012; Alvarez-Ordóñez et al., 2013; Elhadidy and Mohammed, 2013). Information available in the literature so far suggests that the ability to survive in stressful conditions varies substantially among isolates within a given genotype, whereas there is controversy on the fact of whether some genotypes are better equipped to face the challenge of a changing environment. In a study that was undertaken to compare resistance to different processing treatments (high-pressure processing, heat, ultraviolet, and gamma radiation) among different *E. coli* O157:H7 isolates encoding different antiterminator *Q* gene alleles present upstream of the Shiga toxin gene, Malone et al. (2007) showed that isolates encoding the *Q*_933_ allele were more sensitive to all processing treatments than were isolates encoding the *Q*_21_ allele. Moreover, the *stx*-negative isolates were more resistant to UV and gamma radiation (but not to heat or pressure) than were isolates encoding either the *Q*_933_ or *Q*_21_ allelles. Comparing the resistance to different acidic conditions of *E. coli* O157:H7 strains belonging to lineage I and lineage II, Saridakis et al. (2004) revealed that lineage I strains were more resistant to volatile fatty acids than lineage II strains after 6 h of challenge. On the other hand, lineage II strains were more resistant to HCl treatment than lineage I strains. A recent study by Lee et al., 2012 evaluated the association between different bacterial genotypes (LSPS-6 and *stx*) and resistance patterns to different stressors (acid, freeze-thaw, heat, osmotic, oxidative, and starvation) and showed that lineage II strains exhibited lower resistance to heat and starvation than lineage I strains.

Microbial adaptation to a certain stress condition is often associated with enhanced protection against other subsequent stress exposures, which is referred to as “cross protection” (Johnson, 2002). Moreover, and in practice, multiple stress resistance is important in the food industry that applies multiple hurdles as a control measure to decrease pathogen survival (Jay et al., 2005). Although in several occasions it has been proposed that STEC strains more resistant to a given stress tend to be more resistant to various other types of inactivation agents (Humphrey et al., 1995; Benito et al., 1999), there are some reported exceptions to this general trend (Hauben et al., 1997; Uhlich et al., 2008). To characterize global stress resistance patterns and identify robust strains/genotypes with high resistance to multiple stresses, *D*-values were subjected to multivariate analysis (PCA) and were also used to calculate a relative stress resistance value. Although it was not possible to find any STEC O157:H7 strain consistently showing the highest resistance to all the different inactivation treatments tested, it was possible to rank STEC O157:H7 strains according to their robustness. PCA analysis basically confirmed the prior findings in that it clustered together strains of lineage II [all of them of *tir* (*255A*) and non-clade 8], with a multiple stress-sensitive phenotype (sensitive to acid, heat, cold, and starvation stresses) but significantly more resistant to oxidative stress, and separated them from strains of lineages I and I/II. Lineage I/II is an intermediate lineage that has been reported to be more commonly associated with human illness than lineage II and includes strains of the hyper-virulent STEC group responsible for a multistate outbreak linked to spinach (Ziebell et al., 2008; Zhang et al., 2010). Similarly, clade 8 strains have demonstrated higher virulence and association with severe disease outcome than other clades. Also, some investigators have previously suggested that *tir* (*255T*) harboring strains are more virulent for humans than *tir* (*255A*) harboring strains (Bono et al., 2007; Franz et al., 2012; Mellor et al., 2013). In fact, the *tir* (*255T*) genotype has been suggested to be the most distinctive genotype for the detection of bacterial clones with potential risk for human illness from food sources (Elhadidy et al., 2015a) and has been proposed as an attractive candidate to be used as a surrogate marker for tracking highly severe STEC infections (Elhadidy et al., 2015b). Overall, these results suggest that the increased prevalence of *E. coli* O157:H7 illness observed among lineage I/II, *tir* (*255T*), and clade 8 genotypes might be related to the greater stress robustness of these genotypes, a characteristic that likely facilitates transmission of *E. coli* O157:H7 throughout the food chain and influences the disease causing potential of the pathogen. Consistent with our results, Lee et al. (2012) examined the behavior of various clinical and bovine strains of *E. coli* O157:H7 against six stressors commonly found in the food chain and the environment and concluded that some genotypes of STEC O157 associated with human illness had greater multiple stress resistance than did strains of other genotypes. Nonetheless, the study by Lee et al. (2012) was limited to two assays of genotypic characterization (LSPA6 and *stx* genotypes) without accounting for other genotypes used for comparison in the current study [Shiga-toxin-encoding bacteriophage insertion site assay, clade typing, *tir* (*A255T*) polymorphism, and antiterminator *Q* gene allele analysis].

This study focused on genotypic markers that have been suggested as being clinically relevant among *E. coli* O157:H7 with the aim of evaluating whether variations in transmission and/or virulence potential among *E. coli* O157:H7 strains could be attributed, at least in part, to variations in their resistance to stress. Nevertheless, other different genetic features, such as the presence or functionality of genes involved in the general stress response (e.g., *rpoS*) can also impact the phenotypic behavior of strains. Indeed, previous studies have shown that interstrain variability in stress resistance within and between genotypes/serotypes may be also influenced by other genetic traits, such as the status of the general stress response regulator RpoS (Alvarez-Ordóñez et al., 2013).

## Conclusion

This study assessed the resistance of 33 STEC O157:H7 strains to various food-related stresses. Our results showed that strains more commonly associated with human disease were more resistant to food-related stresses highlighting the influence of stressors in the transmission of this human pathogen. Our findings also contribute to increase the knowledge on the resistance of this pathogen to stressors commonly encountered in the food chain, which can lead to the development of new strategies to control the risk of food-borne illness by implementing different decontamination measures in the food processing industry.

## Author Contributions

Concieved and Designed the Experiments: ME, AA-O. Performed the experiments: ME, AA-O. Analyzed the data: ME, AA-O. Wrote the manuscript: ME, AA-O.

## Conflict of Interest Statement

The authors declare that the research was conducted in the absence of any commercial or financial relationships that could be construed as a potential conflict of interest.
