# Supplementary material for: Diversity of Survival Patterns among Escherichia coli O157:H7 Genotypes Subjected to Food-Related Stress Conditions
Source: Front Microbiol. 2016 Mar 15;7:322. doi: 10.3389/fmicb.2016.00322 (PMC4791531; doi:10.3389/fmicb.2016.00322)
Supplement: Supplementary file 1 [file Table_1.PDF]

9 Supplementary material

Table S1. Principal component analysis of D-values for the seven stress resistance assays

**Variable**	**Factor loading in 1st PC**	**Factor loading in 2nd PC**
Acid	-0.19	-0.35
Heat	-0.26	-0.25
Oxidative	0.27	-0.29
Freeze-thaw	0.04	-0.43
Cold	-0.31	0.30
Osmotic	-0.05	-0.33
Starvation	-0.38	-0.12
