# Supplementary material for: Diversity of Survival Patterns among Escherichia coli O157:H7 Genotypes Subjected to Food-Related Stress Conditions
Source: Front Microbiol. 2016 Mar 15;7:322. doi: 10.3389/fmicb.2016.00322 (PMC4791531; doi:10.3389/fmicb.2016.00322)
Supplement: Supplementary file 2 [file Table_2.PDF]

9 Supplementary material

Table S2. Differences in PC scores among STEC O157:H7 genotypes

Genotype	PC score (mean ± SD) in 1st PC	PC score (mean ± SD) in 2nd PC
Lineage I	-0.13±0.00	1.09±0.00
Lineage I/II	-0.65±0.95	-0.07±1.03
Lineage II	2.35±0.95	0.09±1.84
Q_{933}	-0.76±0.87	-0.44±0.95
Q_{21}	0.58±1.89	0.37±1.37
Q_{933}+Q_{21}	0.26±0.55	0.23±1.21
stx_{2a}	-0.33±1.20	-0.30±1.11
stx_{2c}	0.06±1.94	0.04±1.43
stx_{2a+C}	0.39±1.08	0.39±0.85
SBI 1	-0.47±1.03	-0.13±1.11
SBI 3	-0.13±0.00	1.09±0.00
SBI 5	2.37±1.04	-0.28±1.71
SBI 6	-0.76±1.48	0.41±1.15
SBI 21	-0.96±0.00	1.19±0.00
tir (*255T)*	-0.70±0.88	0.03±1.06
tir (*255A*)	1.88±1.36	-0.08±1.63
Clade 8	-0.72±0.95	-0.20±0.95
Non-clade 8	1.66±1.36	0.471.64
